# Controlling enzymatic activity by immobilization on graphene oxide

**DOI:** 10.1007/s00114-017-1459-3

**Published:** 2017-03-30

**Authors:** Paulina Bolibok, Marek Wiśniewski, Katarzyna Roszek, Artur P. Terzyk

**Affiliations:** 1 Faculty of Chemistry, Physicochemistry of Carbon Materials Research Group, Nicolaus Copernicus University in Toruń, Gagarin St. 7, 87-100 Toruń, Poland; 2 INVEST-TECH R&D Center, Plaska St. 32-34, 87-100 Toruń, Poland; 3 Department of Biochemistry, Faculty of Biology and Environment Protection, Nicolaus Copernicus University in Toruń, Gagarin St. 7, 87-100 Toruń, Poland

**Keywords:** Graphene oxide, Immobilization, Catalase, Kinetic parameters, Secondary structure alterations

## Abstract

In this study, graphene oxide (GO) has been applied as a matrix for enzyme immobilization. The protein adsorption capacity of GO is much higher than of other large surface area carbonaceous materials. Its structure and physicochemical properties are reported beneficial also for enzymatic activity modifications. The experimental proof was done here that GO-based biocatalytic systems with immobilized catalase are modifiable in terms of catalyzed reaction kinetic constants. It was found that activity and stability of catalase, considered here as model enzyme, closely depend on enzyme/GO ratio. The changes in kinetic parameters can be related to secondary structure alterations. The correlation between enzyme/GO ratio and kinetic and structure parameters is reported for the first time and enables the conscious control of biocatalytic processes and their extended applications. The biological activity of obtained biocatalytic systems was confirmed in vitro by the use of functional test. The addition of immobilized catalase improved the cells’ viability after they were exposed to hydrogen peroxide and tert-butyl-hydroperoxide used as source of reactive oxygen species.

**Electronic supplementary material:**

The online version of this article (doi:10.1007/s00114-017-1459-3) contains supplementary material, which is available to authorized users.

## Introduction

During the last two decades, biocatalysis has become an important technological process making the synthesis of drugs, food, vitamins, and many other chemical compounds more environmentally friendly, economically profitable, and more sustainable than conventional methods (Li et al. 2016; Sheldon 2007; Sheldon and van Pelt 2013).

Independently of many advantages of enzymes, their use on a large industrial scale is limited by several factors, which include inter alia:

(i) Enzyme instability in the working conditions or during storage—it is an innate property of enzymes, because they are designed to ensure the physiological processes in cells and organisms, not the efficacy of large-scale processes (Huisman and Collier 2013; Wang et al. 2009b).
(ii) High costs of enzymatic processes, that are connected with the inability to efficiently separate catalyst from reaction product, to recover the enzymatic protein, and to reuse it repeatedly (Asgher et al. 2014; Betancor and Luckarift 2008; Sheldon 2007).

The above disadvantages can be overcome by enzyme immobilization. It is currently considered the best and most common method of modifying enzyme properties (Forsyth and Patwardhan 2013; Sheldon 2007; Tang et al. 2014). Despite the huge diversity of strategies, the biocatalyst immobilization is still connected with some inconveniences. The fundamental concern and the strategic step in immobilization is the enzyme support selection, because interactions between protein and carrier directly influence the catalyst efficacy as well as its thermal and chemical stability (Bernal et al. 2014; Wang et al. 2009a,b).

Rapid development of nanotechnology, observed in recent decades, has brought a lot of new supports. Nanomaterials, compared to the compounds in microscale, are characterized by high mechanical strength and exceptional surface properties that allow for more effective interactions with different biological molecules (Scidaa et al. 2011). The large surface area of nanocarriers enables immobilization of higher amounts of enzymes when compared to microcarriers (Ansari and Husain 2012; Cipolatti et al. 2014; He et al. 2016; Kim et al. 2008; de Poulpiquet et al. 2014). These properties of nanocarriers have attracted research attention and found novel applications in biocatalysis in the last years.

Referring to such properties as the easy-to-control shape and size, low price when compared to most other materials, as well as relatively high biocompatibility, the materials most often used in biocatalysis are carbon nanomaterials (Bernal et al. 2014; Poulpiquet et al. 2014). To this group belong single-walled and multiwalled carbon nanotubes (Karachevtsev et al. 2011; Lee et al. 2011; Zebda et al. 2011), soot (Poulpiquet et al. 2014), carbon nanoparticles (Szot et al. 2010), graphene (Liang et al. 2013; Zheng et al. 2010), mesoporous carbon foam (Guo et al. 2010; Wang et al. 2009a), and nanodiamonds (Wei et al. 2010).

The most promising material seems to be graphene oxide (GO). It proved to be a perfect support for immobilized proteins and enzymes due to its solubility in water and large surface area with oxygen functionalities. Therefore, the immobilization of proteins does not demand prior modifications of the surface (Li et al. 2016; Zhang et al. 2010). Despite the vast potential of GO applications, studies regarding its impact on the catalytic properties of immobilized enzymes are still limited. On the other hand, the published results are difficult to compare with each other, because either they relate to different enzymes or they are immobilized by various methods. Literature data by Hernandez-Cancel et al. (2015), concerning the immobilization of bilirubin oxidase on GO sheets, are the first studies on this protein, that aim to determine the effect of chemical glycosylation and immobilization on the catalytic properties of the enzyme. It has been shown that the process of glycosylation leads to a decrease in the catalytic properties of bilirubin oxidase while increasing its thermal stability.

Despite numerous examples in the literature referring to the decrease in biocatalytic activity, e.g., by Hernandez-Cancel et al. (2015) or Zhou et al. (2012), recent research has shown that using nanoparticles as enzyme carriers leads to maintaining or even enhancing immobilized enzyme efficiency (Ding et al. 2015; Johnson et al. 2014). The hydrolytic activity of lipase improved by 55% when enzyme was immobilized on GO (Pavlidis et al. 2012). It was also shown that GO nanosheets covered with PEG enhance the activity of trypsin for casein digestion (Jin et al. 2012). Recently, Wei and collaborators (Wei and Ge 2013) have focused on GO influence on the immobilized catalase conformation and therefore on its activity. GO reduced the share of α-helix structure in the enzyme and also increased the content of β-sheet, causing relaxation of the protein backbone and, therefore, the reduction of catalytic ability of catalase. These changes are dependent on the concentration of the carrier used and on the time of interaction.

Summing, nanomaterials are thought to positively act as enhancers of the immobilized enzyme efficiency (Ansari and Husain 2012), which has focused research interest on new nanobiocatalytic systems.

The aim of this work was to verify GO as valuable support for effective enzyme immobilization due to the precise analysis of kinetic and structural parameters of adsorbed enzyme. We show for the first time the existing correlation between catalase/GO (Cat/GO) ratio and kinetic and structural parameters. In our opinion, the results allow for the conscious control of biocatalytic processes and their extended applications.

## Materials and methods

### Chemicals and reagents

#### Graphene oxide

Graphene oxide was synthesized by a modified Hummer’s method (Krishnamoorthy et al. 2013; Remyamol et al. 2013). Briefly, 50 mL of concentrated H_2_SO_4_ was added to graphite flakes (0.175 g). KMnO_4_ (2.25 g) was slowly added to the suspension. The reaction mixture was kept at 25 °C and stirred for 24 h. The oxidation was stopped by adding 5 mL of 30% H_2_O_2_. After that, the mixture was centrifuged (at 8000*×g* for 5 min). The remaining solid material was washed several times with 200 mL of 30% HCl and with 500 mL of water. After each wash, the mixture was centrifuged (at 15,000*×g* for 20 min). The final resulting material was freeze-dried for 24 h. Before use, GO was diluted in deionized water and ultrasonicated for 60 min to obtain GO solution.

The other two carbonaceous materials were described in details elsewhere (Czarnecka et al. 2016; Wiśniewski et al. 2011).

#### Catalase (Cat)

Catalase from bovine liver (EC 1.11.1.6)—tetramer consisting of four equal subunits each of 60 kDa—was purchased from Sigma-Aldrich. For all the experimental procedures, catalase was dissolved in appropriate concentration in 50 mmol/L PBS buffer, pH 7.4.

### Material characterization

Bulk GO sample was characterized by PXD using Philips XPERT Pro θ-2θ and Bruker D8 diffractometers with CuKα1 and CuKα radiation, respectively. Data were collected from 5 ≤ 2θ/≤120, with a step size of 0.0084° 2θ and at a scanning rate of 0.02° min^−1^.

Scanning electron microscopy (SEM) studies were performed with Quanta 3D FEG (EHT = 30 kV) instrument. Samples were placed onto carbon tabs attached to aluminum SEM stubs. All samples were analyzed in the microscope without coating treatment. Atomic force microscopy (AFM) analysis of lyophilized GO films with 1.0 mg_Cat_/mg_C_ adsorbed was performed using a Veeco microscope (Digital Instruments) with an NSG-11 probe (scan size 2–10 μm; scan rate 1 Hz, tapping mode).

### Enzyme adsorption and desorption

Adsorption of Cat was performed at 4 °C in the initial concentration range of 0.1–5 mg_cat_/mL and the constant concentration of GO 0.1 mg/mL. After 48 h of equilibration, each solution was centrifuged at 5000*×g* for 10 min. The concentration of protein in supernatant was measured spectrophotometrically by measuring the area of the 280-nm band (e.g., Stoscheck 1990; Layne 1957) based on calibration factor determined for catalase. The precipitates were subjected to enzymatic activity assay.

Additionally, the enzyme desorption was tested as follows: After centrifugation of adsorbed equilibrated catalytic systems, fresh portion of PBS was added to the precipitates. The amount of protein was measured after 21 days.

### Enzymatic activity and stability

Enzymatic activity of Cat was determined with H_2_O_2_ as substrate at 25 °C by modifying the procedures described in, e.g., Beers and Sizer (1952) and Li and Schellhorn (2007). Briefly, the dose of 0.1 mL of Cat solution in PBS (75 μg/mL) was added to 2.9 mL H_2_O_2_, in concentration range of 1–100 mmol/L. The changes in the absorbance at 240 nm were monitored continuously during 10-min reaction with Jasco V-660 spectrophotometer.

Storage stability study was performed as follows: Native and immobilized Cat was stored at 4 °C in 50 mmol/L PBS buffer, pH 7.4 for 21 days. The aliquots from each preparation (0.1 mL) were taken in triplicates at established time points and analyzed for the remaining activity. The activity of native Cat, determined on the first day, was taken as control (100%) for the calculation of remaining activity.

### In vitro antioxidant activity of biocatalytic systems

Chinese hamster ovary (CHO) cells were obtained from Sigma-Aldrich. Cells were grown in Ham’s F-12 medium containing 10% fetal bovine serum (FBS) at 37 °C in a CO_2_ incubator with 5% of CO_2_. A volume of 25 μL containing approximately 1 × 10^5^ cells was seeded to each well of a 12-well plate 24 h before the experiment was started. Native Cat or Cat/GO complexes (catalase/GO ratio 2:1 and 15:1, respectively) were added to the growing CHO cells in protein concentration of 250 ng/mL together with 500 μM H_2_O_2_ or 100 μM tert-butyl-hydroperoxide (tBuOOH) and incubated for the next 24 h. Both reagents were used as a source of reactive oxygen species. Subsequently, the MTT test, based on the ability to reduce 3-(4,5-dimethylthiazol-2-yl)-2,5-diphenyltetrazolium bromide (MTT), to assess the viability of cells was performed.

### Fitting catalase adsorption by a numerical model

The bimodal Langmuir-Freundlich equation is represented by expansion of equation proposed by Jeppu and Clement (2012) and Umpleby et al. (2001) to bimodal form

1 $$ {Q}_{eq}=\frac{A_1{\left({K}_1 C\right)}^{\frac{1}{n_1}}}{1+{\left({K}_1 C\right)}^{\frac{1}{n_1}}}+\frac{A_2{\left({K}_2 C\right)}^{\frac{1}{n_2}}}{1+{\left({K}_2 C\right)}^{\frac{1}{n_2}}} $$

2 $$ {A}_x={w}_x{Q}_{m, x}, x=1,2 $$

where *Q*_*eq*_ is the amount adsorbed at equilibrium (mg_Cat_/mg_C_), *Q*_*m*_ is the maximum adsorbed capacity of the system (mg_Cat_/mg_C_), *w*_*x*_ is the weight, *C* is the protein concentration in solution at equilibrium (mg/mL), *K*_*x*_ (*x* = 1,2) is the affinity constant for adsorption (mL/mg), and *n* is the index of heterogeneity.

### Statistical analysis

All experiments were repeated at least three times, and the qualitatively similar results were obtained. The presented results are representative for three tested series of experiments.

## Results

### GO characterization

**Fig. 1:**
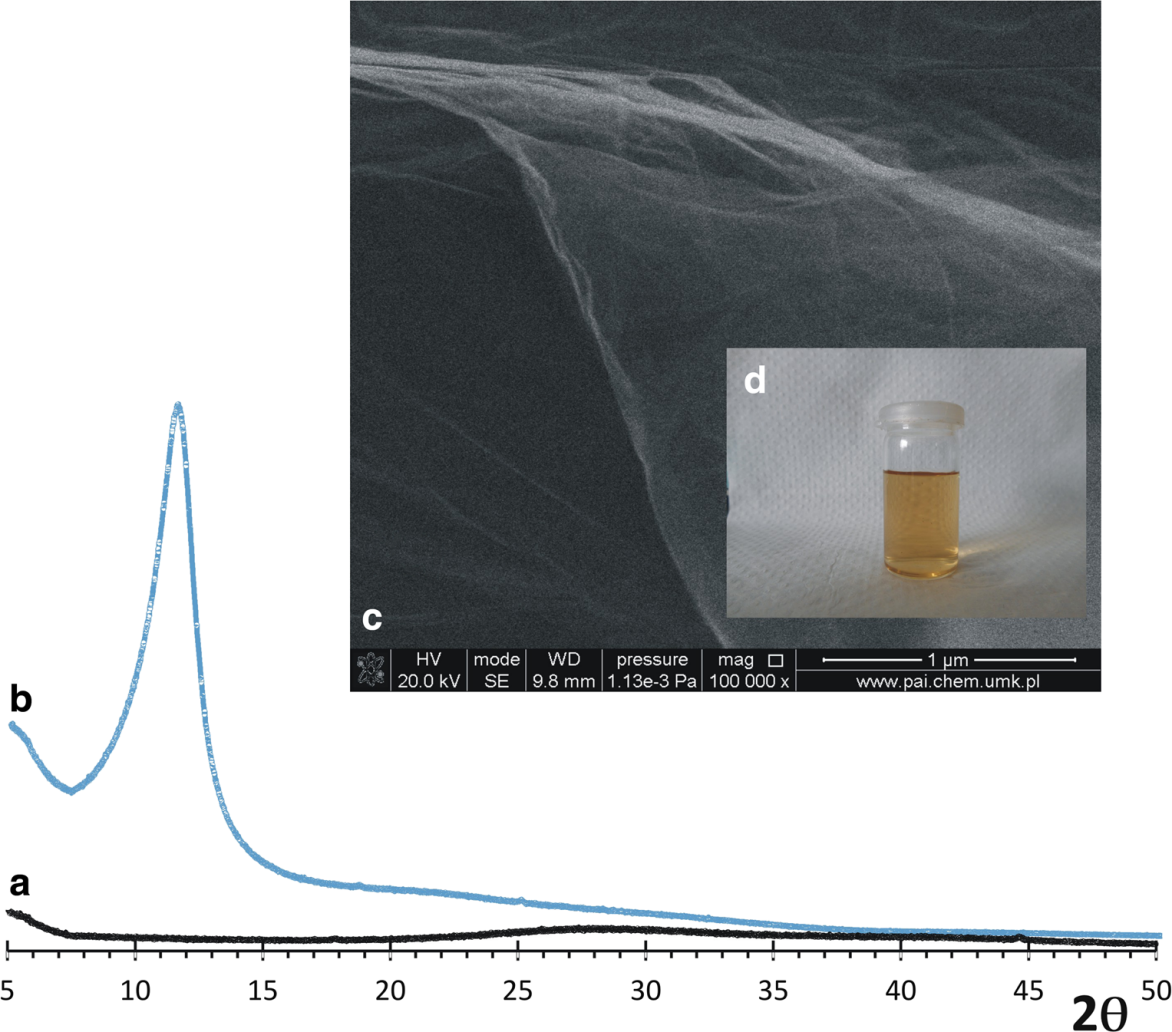
XRD pattern of GO. Slurry after centrifugation (*A*) and after drying the sample (*B*). SEM pictures of obtained (*B*) sample (*C*). The GO solution in concentration of 0.1 mg/mL (*D*)

The modified Hummer’s method allowed for synthesis of homogenous, large-sized GO sheets as shown in Fig. 1. GO large size effects in the formation of numerous folds created due to the strong interaction of the surface functionalities. It is important to note that the single sheets are almost totally transparent, and after dispersion in water, the brownish and stable solution is formed, meaning that each layer exists separately. To confirm this, the XDR analysis was performed (Fig. 1a, b). No signal was present in the spectrum of the centrifuged, non-dried sample (Fig. 1a). After drying, the only signal, appearing at 2θ 11.66°, comes from typical GO sheet aggregates which are separated 0.758 nm based on Bragg’s equation. The above results confirm the presence of detached GO layers in the solution. It is worth to note that there are no signals of graphite.

### Catalase adsorption

The adsorption isotherms for Cat immobilized on GO and for comparison on two other carbonaceous materials have been performed (Fig. S1). CP3 represents high-surface-area carbon materials, whereas CS1 hydrothermal ones. It is clear that GO possesses drastically higher adsorptive capacity than two other carbonaceous materials. Bearing in mind that dry GO measured surface area is lower than 1 m^2^/g (based on N_2_ low-temperature adsorption), while of the other materials, 2130 and 120 m^2^/g for CP3 and CS1, respectively, it is obvious that after dispersion in water, the graphene layers are separated facilitating such high adsorption ratio of proteins.

The bimodal Langmuir-Freundlich equation (Eq. 1) fits well the experimental data. This simple model was presented as applicable to the description of different adsorption data, see, e.g., Huang et al. (2014b). In fact, relatively good fit, i.e., the values of determination coefficient in the range of 0.9966–0.9998 were obtained. The lowest value was obtained for Cat adsorption on CP3 sample, while the highest one for adsorption on GO. Figure S1 shows the quality of the fit for all tested systems, as well as the plots of the individual components of the bimodal model.

The fitted values of *K* and *n* are summarized in Table 1. The constant *K* is related to the affinity between the adsorbate and the adsorbent. The *n* parameter is the site heterogeneity index. Value of *n* = 1 suggests non-interacting sites, while 0 < *n* < 1 the positive cooperativity, whereas *n* > 1, negative cooperativity is expected during adsorption process.

**Table 1:** Fitted parameters of bimodal Langmuir-Freundlich equation

Sample	*K*_1_ (mL/mg)	*n*_1_	*K*_2_ (mL/mg)	*n*_2_	*R* ^2^
GO	1.170	0.417	0.158	2.518	0.9998
CP3	0.326	1.547	0.087	0.158	0.9966
CS1	2.958	0.667	0.094	0.134	0.9986

*R* ^2^ is the value of determination coefficient showing the quality of the fit of theoretical model to experimental data

The left part of the table describes the sites possessing higher affinity of carbon surface to protein. Moreover, for GO and CS1 samples, the positive cooperativity is observed. Oppositely, lower carbon-protein affinity with negative cooperativity for GO, but with positive one in the case of two other samples, is located in the right side of the table.

For GO, found *n* > 1 indicates that negative cooperative adsorption takes place, probably due to steric hindrance caused by multilayer adsorption (see results presented in Fig. 3) rather than to non-favorable electrostatic interactions. Oppositely (for *n* < 1 low enzyme loading) as a result of non-specific adsorption, the protein molecule has a high degree of freedom in movement, both rotational and lateral, which can lead to the increase of enzymatic activity (see also Fig. 4 and Table 2).

**Table 2:** Kinetic parameters of native and immobilized catalase

Catalase amount (mg_Cat_/mg_C_)	*V*_max_ (μmol/min/mg)	*K*_*m*_ (mM)	*V*_max_ */K*_*m*_ (min^−1^)
Native	1568.01	3.44	0.01139
1.34	4756.24	10.68	0.01113
2.16	3976.14	32.28	0.00308
4.36	2569.04	34.27	0.00187
6.22	2128.79	2.91	0.01829
14.39	1603.21	0.78	0.05152

Because of low protein adsorption capacity of CP3 and CS1 samples, we focused only on GO for further immobilization experiments.

**Fig. 2:**
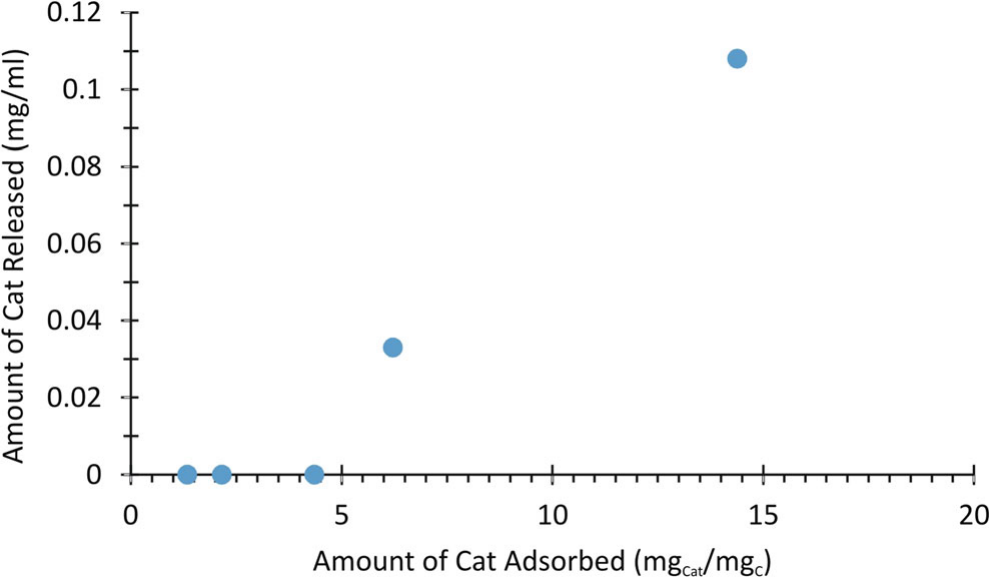
Immobilization stability of tested biocatalytic systems after long-term (21 days) storage at 4 °C in PBS

In order to check the immobilization stability and adsorption reversibility of tested biocatalytic systems, samples with known amount of Cat on GO were centrifuged, and after removal of supernatant, fresh portion of PBS was added to keep the initial GO concentration. The protein concentrations in the solution after 21-day experiment are shown in Fig. 2. One can conclude that for Cat/GO ratio <6, the tested systems are stable, meaning that protein adsorption is completely irreversible. On the contrary, for Cat/GO ratio >6, the adsorption process seems to be reversible.

**Fig. 3:**
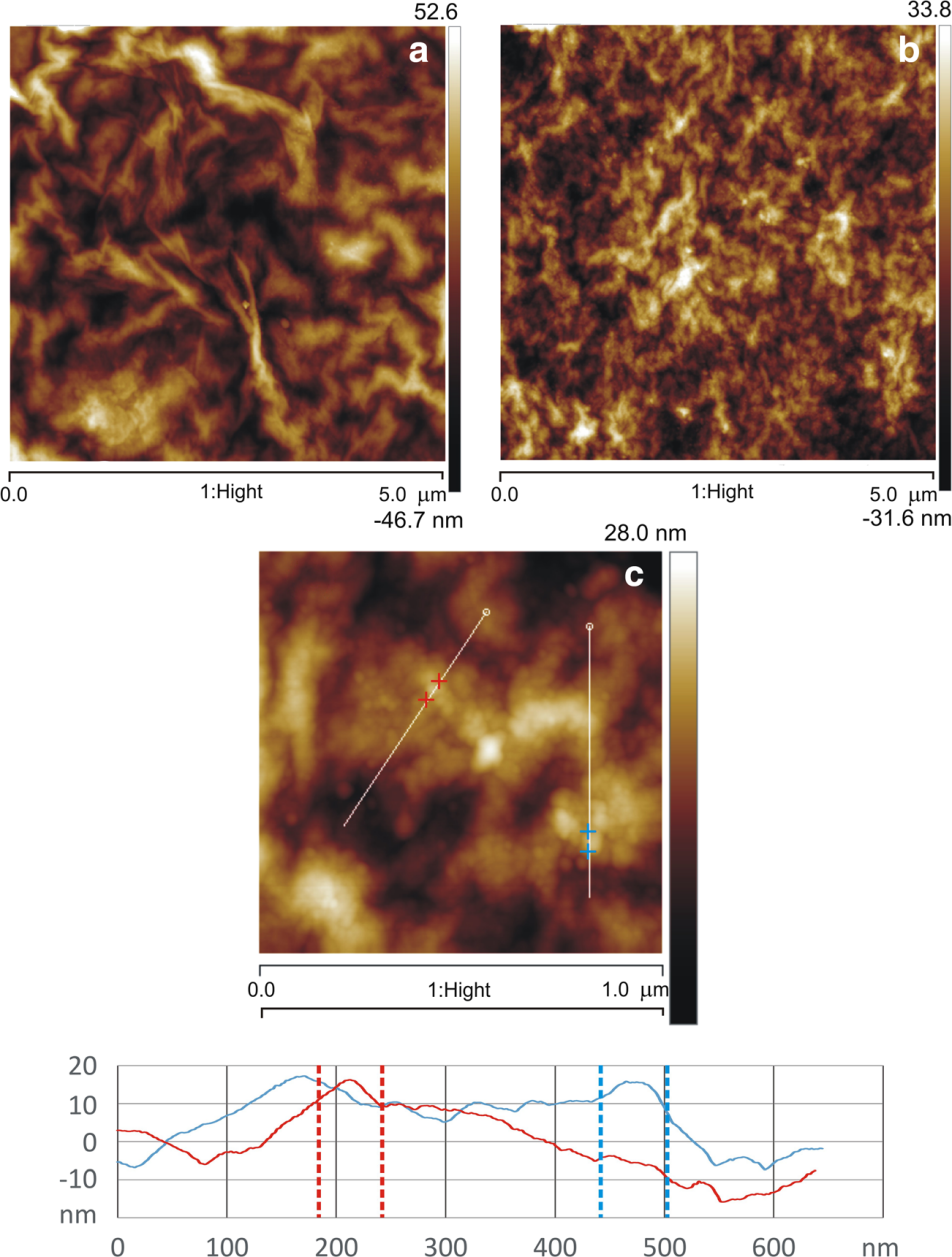
AFM images of GO (**a**) and catalase immobilized on GO (**b**, **c**) with line profile (*bottom*)

The results from AFM analysis are collected in Fig. 3. From careful analysis, one can conclude that there are no drastic changes in the surface structure caused by enzyme immobilization. It means that enzyme subunits are located flat on the surface of GO. The protein on the GO surface forms flat, round structures of ca. 50 nm in diameter and 5–7 nm high.

### Catalase activity and secondary structure

Table 2 summarizes the kinetic properties of studied biocatalytic systems in comparison to the native catalase. The enzymatic reaction kinetic constants are unquestionably related to enzyme/GO ratio. For the biocatalysts with Cat/GO ratio lower than 6, a significant increase in the maximum velocity (*V*_max_) of reaction catalyzed by immobilized enzyme is observed. The simultaneously increasing *K*_*m*_ value correlates with the decreased enzyme affinity to the substrate. The rise in Cat/GO proportion above 6 maintains the reaction velocity slightly higher than native enzyme but also significantly decreases the *K*_*m*_ value, which increases the enzyme affinity to the substrate.

As the modifiable catalytic activity and substrate specificity should have biological consequences, we have tested the functionality and biological activity of obtained Cat/GO complexes, expressed as their antioxidant capacity in the in vitro model. The antioxidant capacity in our experiment is defined as the ability to improve viability of CHO cells exposed to reactive oxygen species (ROS).

**Fig. 4:**
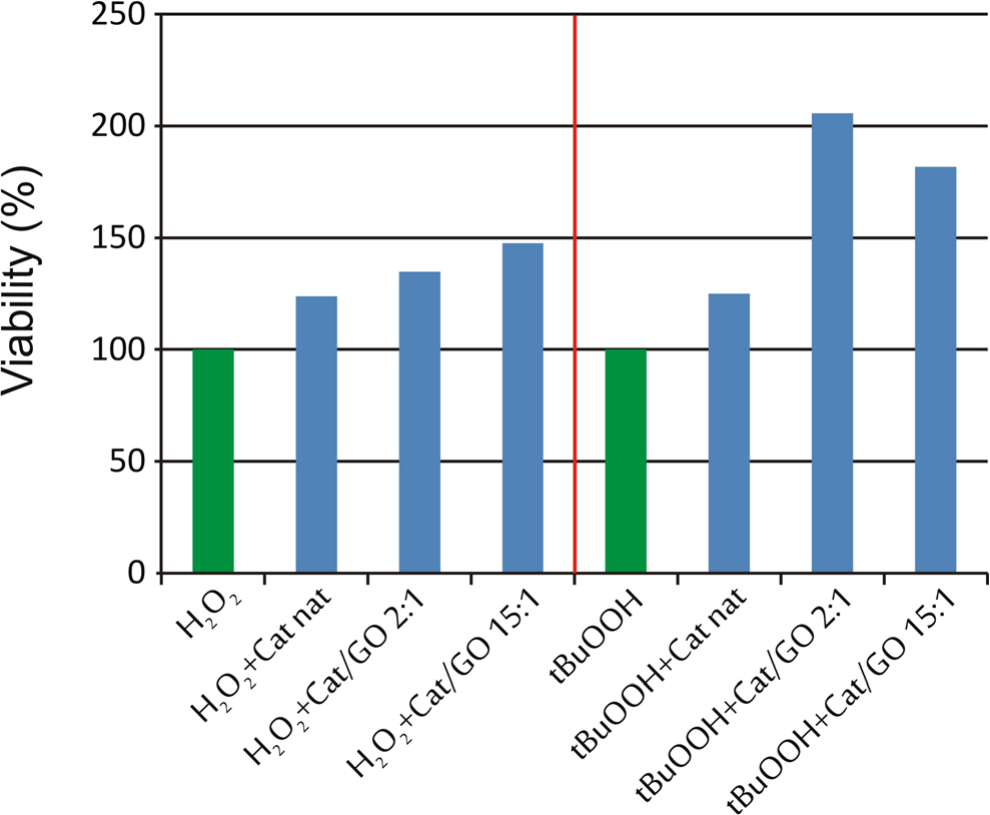
In vitro activity of 250 ng catalase immobilized on GO in ratio 2:1 and 15:1

Figure 4 shows the positive biological influence of catalase, native and immobilized, on GO in ratio 2:1 and 15:1. The native catalase added to culture medium increases the viability of CHO cells by ca. 20%, while catalase immobilized on GO exerts particularly positive effect in the case of tBuOOH-treated cells—their viability increases up to 205% for 2:1 sample.

In order to look closer into the observed phenomena, FT-IR spectroscopy was applied to monitor possible conformational changes of catalase resulting from the enzyme immobilization on GO. The analysis of the amide I band at approximately 1700–1600 1/cm gives information on the immobilization influence on the protein secondary structure (Tzialla et al. 2010). This band consists of several overlapping components that are recognized already after analysis of the second derivative or self-deconvolution (Fig. S2). The bands are assigned to different secondary structure elements (α-helix, β-sheets, β-turns, random coil) (Natalello et al. 2005; Zhao et al. 2012). The spectral decomposition (Fig. S3) of native and immobilized enzyme on GO shows some major changes in the secondary structures that are summarized in Table 3.

**Table 3:** Changes in the secondary structure elements of catalase as a result of protein immobilization on GO

Catalase amount (mg_Cat_/mg_C_)	α-Helix	β-Sheet	Random coil	β-Turn
Native	0.339	0.155	0.165	0.184
14.39	0.322	0.161	0.184	0.182
6.22	0.339	0.129	0.184	0.213
4.36	0.333	0.126	0.180	0.225
2.16	0.312	0.122	0.176	0.256
1.34	0.304	0.118	0.170	0.274

**Fig. 5:**
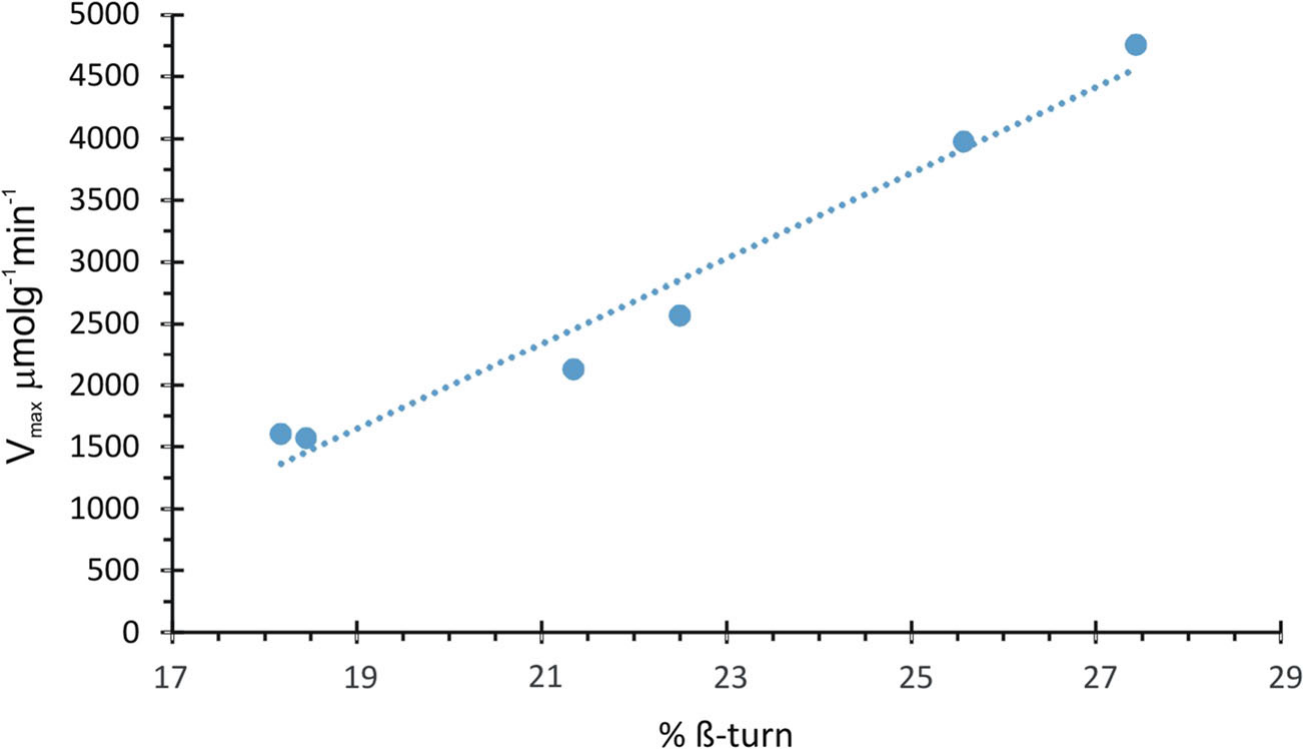
Dependence of immobilized catalase reaction velocity on β-turn contents

Figure 5 presents quantitative relation of kinetic parameter with the structural one. In the literature (Bai et al. 2012), β-turns are considered as special structures formed by reorganizing of some amino acid residues. Therefore, the described results stay in good agreement with the ones from adsorption data and mean that due to non-specific adsorption, the protein molecules have a high degree of freedom in movement, which can lead to the reorganization of secondary structure and to increase of enzymatic activity.

### Catalase storage stability

**Fig. 6:**
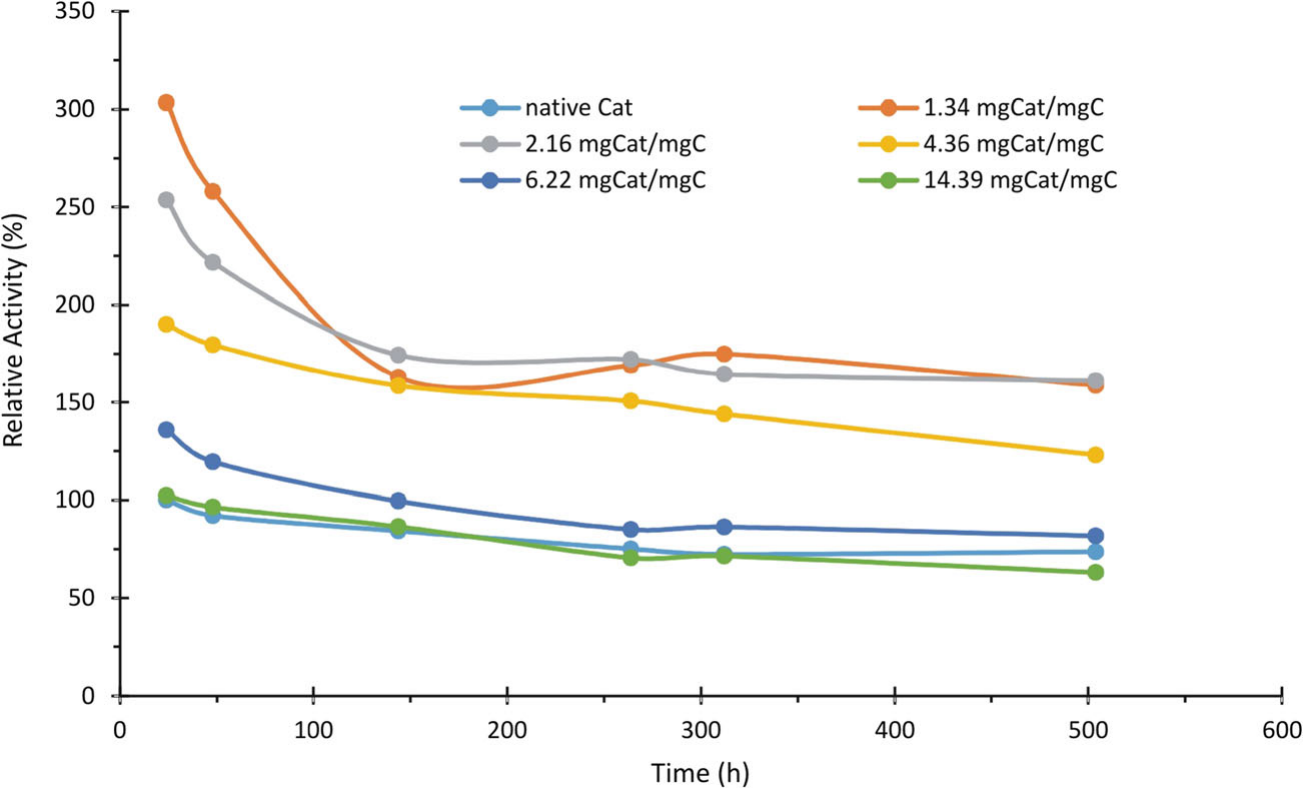
Decay of catalase activity during the storage at 4 °C

Figure 6 summarizes the relative catalytic activity of all tested samples in accordance to their storage stability. It is clear that the presence of two groups of activity, with small and large amount of Cat immobilized, is observed. Cat immobilized on GO with enzyme/GO ratio lower than 6 exhibits high initial activity that decreases rapidly in time and reaches its stable activity (still about 1.5 times higher than native enzyme) after ca. 200 h of storage. Cat immobilized on GO with enzyme/GO ratio higher than 6 reveals activity that gradually decreases from 135 to 105% of initial native Cat activity to the level below 100%.

## Discussion

As it was indicated in the literature (Patila et al. 2016), GO having plenty of oxygen-containing groups on their surface can interact strongly by electrostatic interactions with the protein molecules leading to a very stable conjugates. Presented here results (Fig. 2) confirm this hypothesis. The observed phenomena of different stability can be explained with strong interactions of enzyme with the GO surface particularly evident for low Cat/GO ratio. The adsorption irreversibility of biomolecules on GO was also reported by Varghese et al. (2009) and recently by Deng et al. (2016).

The enzymatic reaction kinetic constants are also related to immobilization process and depend on the enzyme/GO ratio. For the biocatalysts with Cat/GO ratio lower than 6, we obtain a very active enzyme with a lower specificity. When Cat/GO rised over 6, the biocatalytic system with activity similar to the native enzyme but with much higher substrate affinity is achieved. It allows for the controlled alterations of the enzymatic reaction kinetics and for fitting the enzyme parameters to its application.

Catalases, native and immobilized on GO, in ratio 2:1 and 15:1, were tested for its antioxidant action in in vitro models. The first Cat/GO sample (2:1) is characterized by higher enzymatic activity and higher *K*_*m*_ value (see Table 2) that means lower enzyme affinity to substrate than Cat/GO 15:1 sample as well as native enzyme. Since it also has significantly changed structural parameters when compared to the native enzyme (see Table 3), we assume that this biocatalytic system has increased substrate specificity. On the other hand, the sample Cat/GO 15:1 that is structurally and kinetically similar to native enzyme also presents the improved biological activity, when compared to the native catalase.

Studies on the immobilized Cat activity in in vitro models are uncommon in the literature. Batrakova et al. (2007) confirmed the antioxidant capacity of the catalase nanoformulations on microglial ROS production in vitro. Recently, the protective effect of Cat-immobilized nanofibrous mats against H_2_O_2_-induced toxicity was assessed in human umbilical vascular endothelial cells (HUVEC) with or without pretreatment with nanofibrous mats (Huang et al. 2014a). Pretreatment of immobilized catalase reduced the cytotoxicity and protected cells against hydrogen peroxide-induced cytotoxic effects which were demonstrated by MTT assay.

It is clear from the literature (Zámocký and Koller 1999) that for typical catalases, their substrates, while reaching the active site, have to go through the main, hydrophobic, very narrow channel, and therefore, only diffusion of small-sized molecules with low polarity is allowed. Quite different situation is in the case of catalase peroxidases, in which the active sites are easily accessible for all potential substrates. Taking into accordance the presented in this work results, one can postulate that after immobilization of catalase on GO, the active center is readily available not only for H_2_O_2_ but also for other substrates, causing the peroxidase reactions to become significant. Thus, the structural alterations could be responsible for changes in both activity as well as substrate specificity of the process.

Our results concerning the native Cat composition stay in agreement with the secondary structures of bovine Cat analyzed with electron crystallography or X-ray diffraction methods. These structures deposited in Protein Data Bank^1^ reveal that the enzyme consists of 27–32% of α-helices and 14–18% of β-sheet structures, depending on the method used.

As a result of immobilization, we observed that the α-helix content decreases from 34 to 30% and also the β-sheet content decreases from 16% down to 12% depending on the Cat/GO ratio. The decreases in the content of α-helices due to the immobilization are known in the literature (Patila et al. 2016; Wei and Ge 2013). However, the decreasing of β-sheet content of Cat on the GO is not described. Moreover, the increase of the content of β-turns seems to be a novelty for Cat immobilized on GO. Similar results have been recently reported for horseradish peroxidase immobilized on GO (Zhang et al. 2015). Unfortunately, the literature data referring to the enzymatic reaction kinetic constants before and after immobilization on carbonaceous materials are uncommon (e.g., Ansari et al. 2015; Zhang et al. 2013). Nevertheless, De et al. (2011) described the interaction between GO and chymotrypsin. The authors found that GO strongly inhibited the activity of chymotrypsin, which was affected by the coexistence of anionic, hydrophobic, and π-π stacking interactions.

The conformational changes of the enzymes induced by immobilization on other non-carbonaceous materials are described and usually cause decrease of the substrate affinity which is observable as increase of *K*_*m*_. However, it is not a universal principle since there are some literature reports opposing this rule. For example, for laccase used in textile wastewater treatment, *K*_*m*_ value after immobilization increases approximately 20-fold (Cristovao et al. 2012), whereas for glucose oxidase used in estimation of glucose level, immobilization decreased the *K*_*m*_ value by four times (Hashemifard et al. 2010). For a comprehensive review, see Singh et al. (2013) and references therein.

Furthermore, a partial inactivation of all, or the complete inactivation of a part of, the enzyme molecules may occur (decrease of *V*_max_). It was reported that the difference in kinetics between lipases immobilized on different supports was ascribable to conformational changes induced upon enzyme-matrix interaction (Hanefeld et al. 2009). Our results confirm these statements only for high Cat/GO ratio (above 6), whereas enzyme/GO ratio lower than 6 leads to obtaining more active biocatalytic system even after long-term storage. These observations are confirmed by the desorbed protein content assay (Fig. 2).

## Conclusions

The present study provides important new insights into the changes of kinetic parameters attained from the structural alterations of catalase immobilized on GO. Our findings reveal that due to Cat adsorption on GO, the percentage of α-helical and β-sheet structures decreases considerably for Cat/GO ratio lower than 6. The relaxation of secondary structure is followed by the increase in the β-turn region content. The latter, in our opinion, leads to the increase in the catalytic activity. The enzymatic reaction kinetic constants are unquestionably related to Cat/GO ratio. For low enzyme to surface concentration, we obtain a very active enzyme with a lower affinity. Oppositely, for high Cat/GO ratio, the biocatalytic system with activity similar to the native enzyme but with much higher substrate affinity is achieved. It allows for the controlled change in the enzymatic reaction kinetics and for fitting the enzyme parameters to its application. We also confirmed the biological activity of the obtained biocatalytic systems.

## Electronic supplementary material

ESM 1 (DOCX 495 kb)

## Footnotes

1
http://www.rcsb.org/pdb/results/results.do?qrid=68AD8026&tabtoshow=Current

## References

[CR1] Ansari SA, Husain Q (2012). Potential applications of enzymes immobilized on/in nano materials: a review. Biotechnol Adv.

[CR2] Ansari SA, Satar R, Zaidi SK, Naseer MI, Karima S, Alqahtani MH, Rasool M (2015). Nanodiamonds as an effective and novel matrix for immobilizing β galactosidase. Food Bioprod Process.

[CR3] Asgher M, Shahid M, Kamal S, Iqbal HMN (2014). Recent trends and valorization of immobilization strategies and ligninolytic enzymes by industrial biotechnology. J Mol Catal B Enzym.

[CR4] Bai W, Yang Y-J, Tao X, Chenb J-F, Tan T-W (2012). Immobilization of lipase on aminopropyl-grafted mesoporous silica nanotubes for the resolution of (R, S)-1-phenylethanol. J Mol Catal B Enzym.

[CR5] Batrakova EV, Li S, Reynolds AD, Mosley RL, Bronich TK, Kabanov AV, Gendelman HE (2007). A macrophage-nanozyme delivery system for Parkinson’s disease. Bioconjug Chem.

[CR6] Beers RF, Sizer IW (1952). A spectrophotometric method for measuring the breakdown of hydrogen peroxide by catalase. J Biol Chem.

[CR7] Bernal C, Escobar S, Wilson L, Illanes A, Mesa M (2014). Carbonaceous-siliceous composite materials as immobilization support for lipase from *Alcaligenes* sp.: application to the synthesis of antioxidants. Carbon.

[CR8] Betancor L, Luckarift HR (2008). Bioinspired enzyme encapsulation for biocatalysis. Trends in Biotechnol.

[CR9] Cipolatti EP, Silva MJA, Klein M, Feddern V, Feltes MMC, Oliveira JV, Ninow JL, Oliveira D (2014). Current status and trends in enzymatic nanoimmobilization. J Mol Catal B Enzym.

[CR10] Cristovao RO, Silverio SC, Tavares AP, Brigida AI, Loureiro JM, Boaventura RA, Macedo EA, Coelho MA (2012). Green coconut fiber: a novel carrier for the immobilization of commercial laccase by covalent attachment for textile dyes decolourization. World J Microbiol Biotechnol.

[CR11] Czarnecka J, Gołembiewska E, Roszek K, Terzyk AP, Wiśniewski M (2016) Looking for proper toxicity evaluation of carbon nanomaterials (under review)

[CR12] De M, Chou SS, Dravid VP (2011). Graphene oxide as an enzyme inhibitor: modulation of activity of α-chymotrypsin. J Am Chem Soc.

[CR13] Deng X, Chen M, Fu Q, Smeets NMB, Xu F, Zhang Z, Filipe CDM, Hoare T (2016). A highly-sensitive immunosorbent assay based on biotinylated graphene oxide and the quartz crystal microbalance. ACS Appl Mater Interfaces.

[CR14] Ding S, Cargill AA, Medintz IL, Claussen JC (2015). Increasing the activity of immobilized enzymes with nanoparticle conjugation. Curr Opin Biotechnol.

[CR15] Forsyth C, Patwardhan SV (2013). Controlling performance of lipase immobilised on bioinspired silica. J Mater Chem B.

[CR16] Guo CX, Hu FP, Lou XW, Li CM (2010). High-performance biofuel cell made with hydrophilic ordered mesoporous carbon as electrode material. J Power Sources.

[CR17] Hanefeld U, Gardossi L, Magner E (2009). Understanding enzyme immobilization. Chem Soc Rev.

[CR18] Hashemifard N, Mohsenifar A, Ranjbar B, Allameh A, Lotfi AS, Etemadikia B (2010). Fabrication and kinetic studies of a novel silver nanoparticles-glucose oxidase bioconjugate. Anal Chim Acta.

[CR19] He W, Elkhooly TA, Liu X, Cavallaro A, Taheri S, Vasilev K, Feng Q (2016). Silver nanoparticle based coatings enhance adipogenesis compared to osteogenesis in human mesenchymal stem cells through oxidative stress. J Mater Chem B.

[CR20] Hernandez-Cancel G, Suazo-Davila D, Ojeda-Cruzado AJ, Garcia-Torres D, Cabrera CR, Griebenow K (2015). Graphene oxide as a protein matrix: influence on protein biophysical properties. J Nanobiotechnol.

[CR21] Huang R, Deng H, Cai T, Zhan Y, Wang X, Chen X, Ji A, Li X (2014). Layer-by-layer immobilized catalase on electrospun nanofibrous mats protects against oxidative stress induced by hydrogen peroxide. J Biomed Nanotechnol.

[CR22] Huang W, Zhou X, Xia Q, Peng J, Wang H, Li Z (2014). Preparation and adsorption performance of GrO@Cu-BTC for separation of CO_2_/CH_4_. Ind Eng Chem Res.

[CR23] Huisman GW, Collier SJ (2013). On the development of new biocatalytic processes for practical pharmaceutical synthesis. Curr Opin Chem Biol.

[CR24] Jeppu GP, Clement TP (2012). A modified Langmuir-Freundlich isotherm model for simulating pH-dependent adsorption effects. J Contam Hydrol.

[CR25] Jin L, Yang K, Yao K, Zhang S, Tao H, Lee ST, Liu Z, Peng R (2012). Functionalized graphene oxide in enzyme engineering: a selective modulator for enzyme activity and thermostability. ACS Nano.

[CR26] Johnson BJ, Algar WR, Malanoski AP, Ancona MG, Medintz IL (2014). Understanding enzymatic acceleration at nanoparticle interfaces: approaches and challenges. Nano Today.

[CR27] Karachevtsev VA, Stepanian SG, Glamazda AY, Karachevtsev MV, Eremenko VV, Lytvyn OS, Adamowicz L (2011). Noncovalent interaction of single-walled carbon nanotubes with 1-pyrenebutanoic acid succinimide ester and glucoseoxidase. J Phys Chem C.

[CR28] Kim J, Grate JW, Wang P (2008). Nanobiocatalysis and its potential applications. Trends in Biotechnol.

[CR29] Krishnamoorthy K, Veerapandian M, Yun K, Kim S-J (2013). The chemical and structural analysis of graphene oxide withdifferent degrees of oxidation. Carbon.

[CR30] Layne E (1957). Spectrophotometric and turbidimetric methods for measuring proteins. Methods Enzymol.

[CR31] Lee JY, Shin HY, Kang SW, Park C, Kim SW (2011). Application of an enzyme-based biofuel cell containing a bioelectrode modified with deoxyribonucleic acid-wrapped single-walled carbon nanotubes to serum. Enzym Microb Technol.

[CR32] Li Y, Schellhorn HE (2007). Rapid kinetic microassay for catalase activity. J Biomol Tech.

[CR33] Li W, Wen H, Shi Q, Zheng G (2016). Study on immobilization of (+) γ-lactamase using a new type of epoxy graphene oxide carrier. Process Biochem.

[CR34] Liang B, Fang L, Yang G, Hu Y, Guo X, Ye X (2013). Direct electron transfer glucose biosensor based on glucose oxidase self-assembled on electrochemically reduced carboxyl grapheme. Biosens Bioelectron.

[CR35] Natalello A, Ami D, Brocca S, Lotti M, Doglia SM (2005). Secondary structure, conformational stability and glycosylation of a recombinant *Candida rugosa* lipase studied by Fourier-transform infrared spectroscopy. J Biochem.

[CR36] Patila M, Pavlidis IV, Kouloumpis A, Dimos K, Spyrou K, Katapodis P, Gournis D, Stamatis H (2016). Graphene oxide derivatives with variable alkyl chain length and terminal functional groups as supports for stabilization of cytochrome c. Inter J Biol Macromol.

[CR37] Pavlidis I, Vorhaben T, Gournis D, Papadopoulos G, Bornscheuer U, Stamatis H (2012). Regulation of catalytic behaviour of hydrolases through interactions with functionalized carbon-based nanomaterials. J Nanopart Res.

[CR38] Poulpiquet A, Ciaccafava A, Lojou E (2014). New trends in enzyme immobilization at nanostructured interfaces for efficient electrocatalysis in biofuel cells. Electro Acta.

[CR39] Remyamol T, John H, Gopinath P (2013). Synthesis and nonlinear optical properties of reduced graphene oxide covalently functionalized with polyaniline. Carbon.

[CR40] Scidaa K, Stege PW, Habya G, Messina GA, García CD (2011). Recent applications of carbon-based nanomaterials in analytical chemistry: critical review. Anal Chim Acta.

[CR41] Sheldon RA (2007). Enzyme immobilization: the quest for optimum performance. Adv Synth Catal.

[CR42] Sheldon RA, van Pelt S (2013). Enzyme immobilisation in biocatalysis: why, what and how. Chem Soc Rev.

[CR43] Singh RK, Tiwari MK, Singh R, Lee JK (2013). From protein engineering to immobilization: promising strategies for the upgrade of industrial enzymes. Int J Mol Sci.

[CR44] Stoscheck CM (1990). Quantitation of protein. Methods Enzymol.

[CR45] Szot K, Watkins JD, Bull SD, Marken F, Opallo M (2010). Three dimensional film electrode prepared from oppositely charged carbon nanoparticles as efficient enzyme host. Electrochem Commun.

[CR46] Tang C, Saquing CD, Sarin PK, Kelly RM, Khan SA (2014). Nanofibrous membranes for single-step immobilization of hyperthermophilic enzymes. J Membr Sci.

[CR47] Tzialla AA, Pavlidis IV, Felicissimo MP, Rudolf P, Gournis D, Stamatis H (2010). Lipase immobilization on smectite nanoclays: characterization and application to the epoxidation of *α*-pinene. Bioresour Technol.

[CR48] Umpleby RJ, Baxter SC, Chen Y, Shah RN, Shimizu KD (2001). Characterization of molecularly imprinted polymers with the Langmuir−Freundlich isotherm. Anal Chem.

[CR49] Varghese N, Mogera U, Govindaraj A, Das A, Maiti PK, Sood AK, Rao C (2009). Binding of DNA nucleobases and nucleosides with graphene. ChemPhysChem.

[CR50] Wang K, Yang H, Zhu L, Ma Z, Xing S, Lv Q, Liao J, Liu C, Xing W (2009). Direct electron transfer and electrocatalysis of glucose oxidase immobilized on glassy carbon electrode modified with Nafion and mesoporous carbon FDU-15. Electro Acta.

[CR51] Wang Z-G, Wan L-S, Liu Z-M, Huang X-J, Xu Z-K (2009). Enzyme immobilization on electrospun polymer nanofibers: an overview. J Mol Catal B Enzym.

[CR52] Wei X-L, Ge Z-Q (2013). Effect of graphene oxide on conformation and activity of catalase. Carbon.

[CR53] Wei L, Zhang W, Lu H, Yang P (2010). Immobilization of enzyme on detonation nanodiamond for highly efficient proteolysis. Talanta.

[CR54] Wiśniewski M, Pacholczyk A, Terzyk AP, Rychlicki G (2011). New phosphorus-containing spherical carbon adsorbents as promising materials in drug adsorption and release. J Colloid Interface Sci.

[CR55] Zámocký M, Koller F (1999). Understanding the structure and function of catalases: clues from molecular evolution and in vitro mutagenesis. Prog Biophys Mol Biol.

[CR56] Zebda A, Gondran C, Goff AL, Holzinger M, Cinquin P, Cosnier S (2011). Mediatorless high-power glucose biofuel cells based on compressed carbon nanotube-enzyme electrodes. Nat Commun.

[CR57] Zhang C, Chen S, Alvarez PJJ, Chen W (2015). Reduced graphene oxide enhances horseradish peroxidase stability by serving as radical scavenger and redox mediator. Carbon.

[CR58] Zhang C, Luo S, Chen W (2013). Activity of catalase adsorbed to carbon nanotubes: effects of carbon nanotube surface properties. Talanta.

[CR59] Zhang J, Zhang F, Yang H, Huang X, Liu H, Zhang J, Guo S (2010). Graphene oxide as a matrix for enzyme immobilization. Langmuir.

[CR60] Zhao H-Z, Du Q, Li Z-S, Yang Q-Z (2012). Mechanisms for the direct electron transfer of cytochrome c induced by multi-walled carbon nanotubes. Sensors.

[CR61] Zheng W, Zhao HY, Zhang JX, Zhou HM, Xu XX, Zheng YF, Wang YB, Cheng Y, Jang BZ (2010). A glucose/O_2_ biofuel cell base on nanographene platelet-modified electrodes. Electrochem Commun.

[CR62] Zhou L, Jiang Y, Gao J, Zhao X, Ma L, Zhou Q (2012). Oriented immobilization of glucose oxidase on graphene oxide. Biochem Eng J.

