# Supplementary material for: Controlling enzymatic activity by immobilization on graphene oxide
Source: Naturwissenschaften. 2017 Mar 30;104(3):36. doi: 10.1007/s00114-017-1459-3 (PMC5374183; doi:10.1007/s00114-017-1459-3)
Supplement: Supplementary file 1 — (DOCX 495 kb) [file 114_2017_1459_MOESM_ESM.docx]

**Supplementary data**

**for**

**Controlling of enzymatic activity by immobilization on graphene oxide**

Paulina Bolibok, Marek Wiśniewski, Katarzyna Roszek, Artur P. Terzyk

**number of pages: 4**

**number of figures: 3**


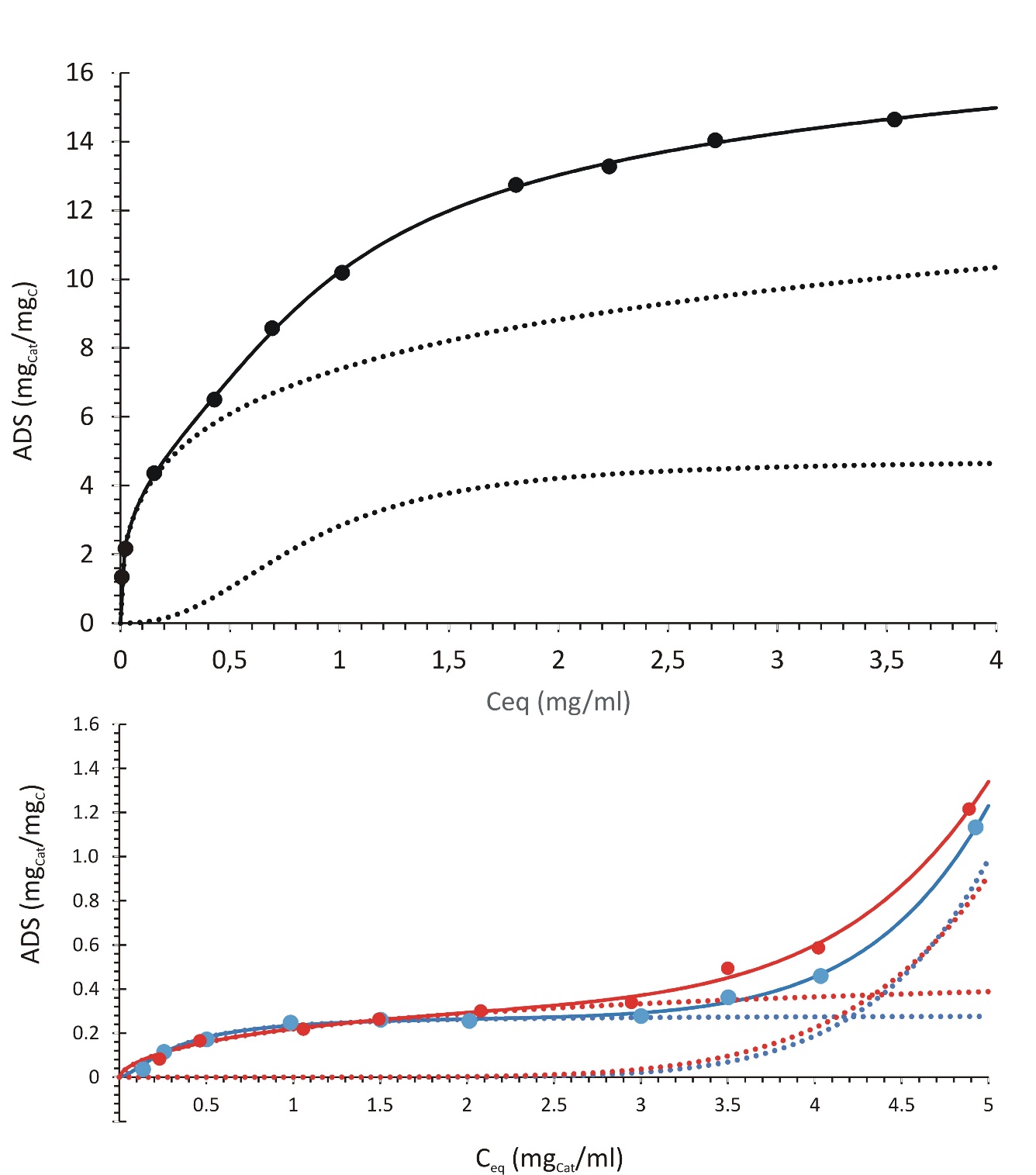


**Figure S1.** Adsorption isotherms of catalase on carbonaceous carriers. Upper panel – GO, bottom – CP3 (red), CS1 (blue).


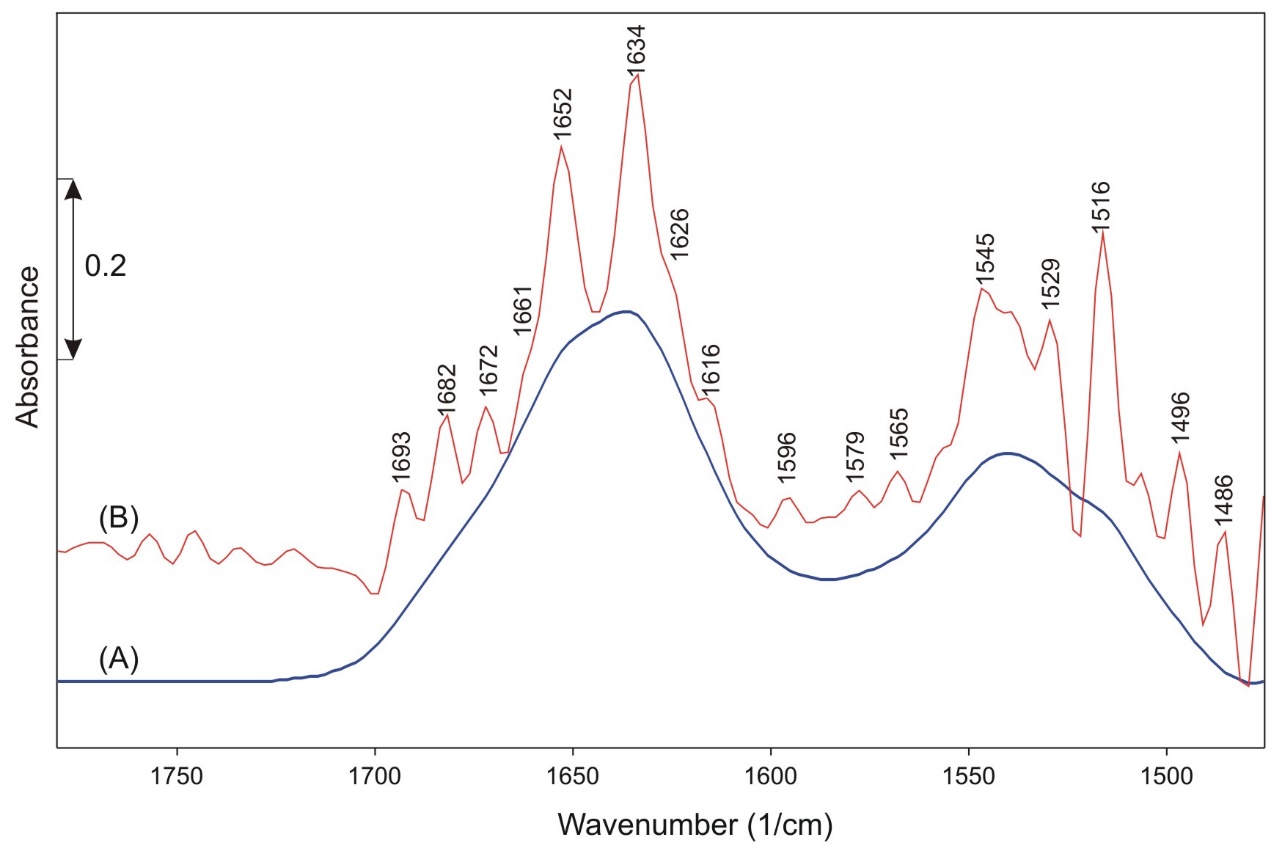


**Figure S2.** The amide I &II region FTIR spectrum of native catalase (A) with self-deconvolution (B).


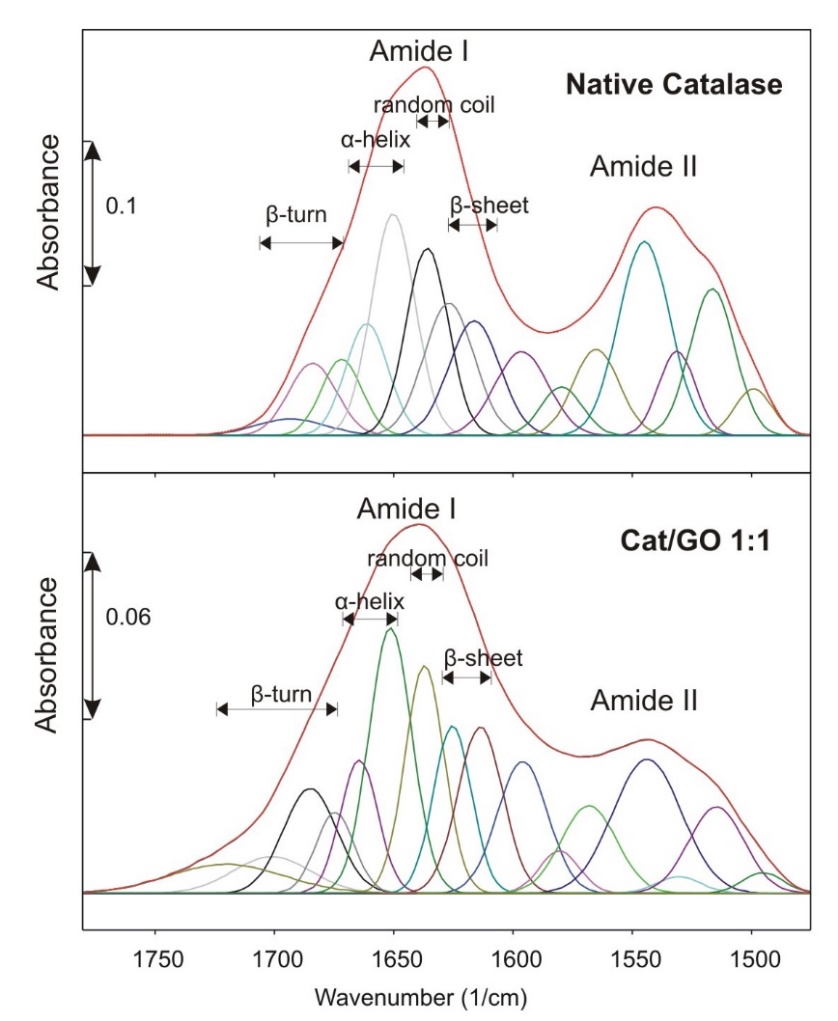


**Figure S3.** The spectral decomposition of amide I & II of native and immobilized enzyme.
